# Prevalence and Effectiveness of Laxative Use Among Elderly Residents in a Regional Hospital Affiliated Nursing Home in Hsinchu County

**DOI:** 10.17795/nmsjournal13962

**Published:** 2014-04-17

**Authors:** I-Chun Chen, Hsiu Ju Huang, Shu Fang Yang, Chia Chi Chen, Yu Chen Chou, Tsam Ming Kuo

**Affiliations:** 1 Department of Physical Medicine and Rehabilitation, Ton-Yen General Hospital, Hsinchu County, Taiwan; 2 Department of Nursing, Chong-De Nursing Home, Hsinchu County, Taiwan; 3 Department of Neurology, National Taiwan University Hospital, Hsin-Chu Branch, Hsinchu County, Taiwan

**Keywords:** Nursing Homes, Constipation, Laxatives

## Abstract

**Background:**

Long-term care residents are susceptible to constipation and one-half to three quarter of older nursing home residents receive laxatives regularly.

**Objectives:**

The purpose of this study was to evaluate the factors related to abnormal bowel function and explore the effectiveness of laxative treatment among the elderly residents of a nursing home.

**Patients and Methods:**

A total of 110 residents older than 65 years old was enrolled in this study. The following variables were gathered: age, gender, body mass index (BMI), length of stay, daily fluid intake, type of food, functional level, cognitive ability, physical therapy status, somatic and psychiatric diseases, number of medications, and medication use. The use and dosage of laxatives were recorded by means of Anatomical Therapeutic Chemical (ATC) classification system. Normal bowel function was defined as defecation frequency from three defecations per day to three defecations per week and stool consistency score of three to five on Bristol Stool Form Scale. A comparison between groups with normal and abnormal bowel function was drawn.

**Results:**

Low BMI, increased fluid intake, liquid food intake, poor functional level, poor cognition, and a history of stroke were significantly associated with altered bowel function (P < 0.05). The most frequently used laxatives were glycerol, senna glycoside, and magnesium oxide. There were significant differences in laxative regimens between residents with normal and altered bowel function; those with altered bowel function tended to take more laxatives than those with normal bowel function.

**Conclusions:**

This study suggested that treatment of constipation in the nursing home was unsatisfactory. To improve treatment outcomes in those susceptible to altered bowel function, a coordinated approach with involvement of physicians, nursing staff, and other professionals including dieticians and pharmacists seems necessary.

## 1. Background

Constipation is a prevalent problem among elderly population. Complaints of constipation and its prevalence increase with age, particularly after the age of 65 (1, 2). The majority of the constipation causes in the elderly are related to the medication use or coexisting medical illnesses; other risk factors of constipation in the elderly population include impaired mobility, low fluid intake, low dietary fiber, and institutionalization (3-10). Long-term care residents are susceptible to constipation and half of them have constipation as a significant problem. Notably, the prevalence of constipation ranges from 44 to 74% in the nursing homes (3, 4, 9, 11-13). The high prevalence of constipation imposes high expenses ranging from laxative expenditure to nursing time (14). In addition to conservative intervention (dietary fiber, physical activity, fluid, etc.), laxatives are the cornerstone of the treatment of constipation. A survey by Goh et al. (15) 2.0% to 5.1% of older people reported the use of laxatives and laxatives were second only to analgesics as the most commonly used over-the-counter (OTC) medication. Another study investigated differences in drug use pattern between community-dwelling and the institutionalized elderly; there were 33.9% of the institutionalized elderly versus 6.2% of the community-dwelling elderly population who reported using laxatives (16). Besides, several studies reported that one-half to three-quarters of the elderly nursing home residents received laxatives regularly (11-13, 17, 18). As a result of the high prevalence of laxative-use in elderly nursing home residents, adverse effects and overuse are the main concerns. Side effects such as abdominal discomfort, electrolyte imbalances, allergic reactions, and hepatotoxicity have been reported (3, 19). Over-prescription of laxatives to the elderly can be attributed to two factors: lack of objective confirmation of the diagnosis and ineffective prescribing patterns (14). In trials conducted in elderly adults, there is little evidence of differences in effectiveness between different categories of laxatives; moreover, a stepped approach to laxative treatment in the elderly people is justified, starting with cheaper laxatives before proceeding to more expensive alternatives (14). In the elderly, use of laxatives must be individualized with special attention to their medical history, drug interactions, costs, and side effects (9). Some studies have investigated the prevalence and associated factors of laxatives use or constipation symptoms among the nursing home elderly population (12, 13, 18, 20, 21). Though some studies reviewed the updated management of constipation and discussed the efficiency of individual laxatives (9, 11, 22), there are limited data on the effectiveness of laxative therapy among elderly nursing home residents (12).

## 2. Objectives

The purpose of this study was to evaluate the factors related to the abnormal bowel function and explore the effectiveness of laxative treatment among the elderly of a nursing home. 

## 3. Patients and Methods

### 3.1. Facility and Residents

The clinical trial was conducted in a certified, professionally run nursing home, which is an affiliated facility of a regional hospital in Hsinchu County, Taiwan, from January to June in 2012. In addition to those from Hsinchu area, many of the residents come from the nearby counties or cities. A physician examined and evaluated all the residents admitted to the nursing home for past medical history, current physical and functional conditions, and prescription of the routine medications. Daily prescribed medication of the residents were formally recorded. From January to June in 2012, residents older than 65 years old in the facility were evaluated. Those who had stayed at the nursing home for more than six months were included in this study. Those who left the nursing home for another institution or returned home were excluded from the study. We further excluded those residents with known organic gastrointestinal diseases such as cancer, inflammatory bowel disease, and intestinal resection. Finally, A total of 110 residents were enrolled in this study. 

### 3.2. Data Acquisition

#### 3.2.1. General Characteristics

The following variables were gathered: age, gender, body mass index (BMI), length of stay, daily fluid intake, type of food (regular, soft, or liquid diet), functional level scored by Barthel Index (23), cognitive ability scored by Mini-Mental Status Examination (MMSE) (24), physical therapy status, somatic and psychiatric diseases, number of medications, and medication use. Barthel Index is one of the best known and most frequently used functional assessment scales; it rates 10 aspect of function, using different relative weights for each variable, with a score ranging from 0 (totally dependent) to 100 (totally independent). It was suggested that scores of 0 to 20 indicate total dependency, 21 to 60 indicate severe dependency, 61 to 90 indicate moderate dependency, and 91 to 99 indicate slight dependency. Mini-Mental Status Examination is a 30-point scale measuring attention, orientation, recall, calculation, and visual perception, which is a brief test for screening the cognitive impairment. Any score≥ 25 points (out of 30) indicates a normal cognition. Scores below that indicate severe (≤ 9 points), moderate (10 - 18 points), or mild (19 - 24 points) cognitive impairment. 

#### 3.2.2. Use of Laxatives

The use and dosage of laxatives was recorded by means of the Anatomical Therapeutic Chemical (ATC) Classification System. Groups of laxatives were defined at ATC-level 5: osmotically acting laxatives (A06AD) such as magnesium oxide and lactulose; contact laxatives (A06AB) such as bisacodyl and senna glycosides; bulk laxatives (A06AC) such as sterculia; enemas (A06AG) such as glycerol and sorbitol; and softeners/emollients (A06AA) such as liquid paraffin. The dosage of each laxative was graded as on demand, regular use of standard dose, and regular use of high dose. High dose was defined as bisacodyl > 10 mg/day; senna glycosides > 24 mg/day; sterculia > 2 package (14GM) per day; lactulose > 30 mL/day; and magnesium oxide > 1000 mg/day (12).

#### 3.2.3. Bowel Function

Defecation frequency (number of stools per day), stool consistency (based on Bristol Stool Form Scale score from type 1 to 7), straining, sensation of incomplete evacuation, sensation of anorectal obstruction, and manual maneuvers to facilitate bowel movements were recorded. Bristol Stool Form Scale can be used to monitor change in intestinal function. The seven types of stool are as follows: type 1, separate hard lumps, like nuts; type 2, sausage-shaped, but lumpy; type 3, like a sausage but with cracks on its surface; type 4, like a sausage or snake, smooth and soft; type 5, soft blobs with clear cut edges; type 6, fluffy pieces with ragged edges, a mushy stool; and type 7, watery, no solid pieces (25). In our study, normal bowel function was defined as defecation frequency from three defecations per week to three defecations per day and a stool consistency score of three to five on the Bristol Stool Form Scale (12). Those residents with normal bowel function were defined as group 1, and those who with altered bowel function were defined as group 2.

### 3.3. Ethical Considerations

This study was performed in compliance with the guidelines for research involving humans and was approved by the institutional review board of the National Taiwan University Hospital Hsin-chu Branch.

### 3.4. Data Analysis

Comparisons between groups were performed with the Independent t-test, the exact chi-square test, and Fisher’s exact test using Statistical Package for the Social Sciences (SPSS; Version 17; SPSS Inc., Chicago, Illinois, USA). In all tests, P-values < 0.05 indicated a statistically significant difference.

## 4. Results

### 4.1. Residents

The study included 110 residents who were older than 65 years and had been continuously staying in the nursing home for more than 6 months. The mean age of the participants was 80.7 ± 7.6 years, ranging from 65 to 99 years. Participants included 55 males and 55 females. The average duration of nursing home stay for all subjects was 40.7 ± 25.7 months, ranging from six to 117 months. The number of diseases of all subjects was categorized and listed as nil in 21 (19.1% of all), one in 49 (44.5% of all), two in 30 (27.3% of all), and three in 10 participants (9.1% of all). There was no significant difference between the group 1 and group 2 in terms of the number of diseases. The number of medications of all subjects was categorized and listed as 0 to 4 in 42 (38.2% of all), 5 to 8 in 49 (44.5% of all), and more than 8 in 19 participants (17.3% of all). There was no significant difference between the group 1 and 2 in the number of medications. Table 1 shows the characteristic variables of the subjects in details.

### 4.2. Predictors of Normal Bowel Function

Table 1 shows the characteristics of the subjects and comparisons between the group 1 and group2. Low BMI, increased fluid intake, liquid food intake, low Barthel Index score, low MMSE score, and a history of stroke were significantly associated with the altered bowel function.

### 4.3. Use of Laxatives

There were 18 (16.4%) participants having no record of laxative use or only using an enema on demand; a total of 92 (83.6%) residents in our study used laxatives regularly. Only nine (8.2%) participants did not take laxatives during the investigation period and all of them had normal bowel function. In other words, 101 (91.8%) participants ever took laxatives during the investigation period. Table 2 demonstrates the dosage schedule of the various laxatives. The most frequently used laxatives, either on demand or regular use, were glycerol, senna glycoside, and magnesium oxide, used by 78 (70.9%), 66 (60%), and 51 (46.4%) participants, respectively. The most frequently regularly used laxatives were senna glycoside, magnesium oxide, and glycerol, used by 66 (60%), 51 (46.4%), and 31 (28.2%) participants, respectively. The glycerol enema was usually used on demand in this study.

### 4.4. Effect of laxatives

Table 3 illustrates that the bowel function was related to the efficacy of the laxative regimen. Significant differences in laxative regimens between group 1 and group2 were found.

## 5. Discussion

Senna glycoside was the most commonly and regularly used laxative in this study (60% in all), and glycerol enema was the most frequently used laxative on demand (42.7% of all). Senna glycoside generally induces evacuation eight to 12 hours following administration and can be taken at bedtime. In addition to the convenience, senna glycoside is a cheap and safe agent for use in the elderly (5). Glycerol enema is widely used for its acute disimpaction effect. For example, some frail elderly residents with poor mobility or neurogenic bowel dysfunction may have recurrent stool impactions despite the regular laxative use, and they often benefit from enemas (14, 26). In contrast to the other studies (12, 18), lactulose was less used by our participants for local therapeutic tradition, because of the rather high cost, and/or the concern of causing abdominal cramps and flatulence. In our study, magnesium oxide was commonly prescribed and even used at high dosage (over 1000 mg/day). Physicians may favor magnesium salts for their rapid action and effects on softening the stool, but the risk of dehydration, fecal incontinence, and hypermagnesemia in the elderly people should be considered (8, 14, 19). In this study, 43 (39%) of the residents treated for constipation did not obtain normal bowel function. The data was similar to another report, showing 41% of residents in nursing homes did not achieve normalization of stool frequency and consistency (12). This was judged as unsatisfactory, although it was at least as good as in clinical trials reporting 40% to 85% of non-responders (27). However, comparisons are difficult because the definitions of satisfactory response vary. We found that a low BMI, low Barthel Index score, low MMSE score, history of stroke, type of liquid food by nasogastric tube feeding, and increased fluid intake were associated with altered bowel function. Previous studies indicated that reduced mobility, loss of functional status, cognitive impairment, past history of stroke, and enteral nutrition were related factors to constipation in elderly people (5, 10, 28-30), and our results were roughly similar to these findings. However, increased fluid intake was associated with altered bowel function in our investigation, which was contradictory to the previous reports (12, 14). An explanation might be that those residents on a liquid diet by tube feeding usually had impaired function and multiple disabilities. Although they had an increased fluid intake, poor functional performance may have played a more significant role in causing altered bowel function. Though some studies indicated that certain medications such as benzodiazepine derivatives, antidepressants, and medications with a markedly anticholinergic effect, were associated with constipation (8-10, 12, 21), our result did not demonstrate any significance with regard to these medications use. Some medical conditions have been mentioned to be related to the constipation, such as diabetes mellitus, Parkinson disease, mood-related disorders, uremia, and spinal cord injury/diseases (8, 14, 31-33). However, our results did not show any significance in these factors. This study revealed significant differences in laxative regimens between participating residents with normal and altered bowel function; in other words, residents with altered bowel function tended to take more laxatives than those with normal bowel function. It suggests that treatment of constipation in the elderly residents is far from acceptable remedies; altered bowel function persisted in some residents, even when two or more than two regular laxatives were taken. These findings agree with a previous conclusion, indicating high rates of self-reported constipation despite the substantial levels of laxatives prescribing in nursing homes. These observations pointed out that non-pharmacological treatments for constipation were underused (5). Consequently, for residents with associated factors of altered bowel function such as impaired self-care and mobility function, poor cognition, tube feeding, and stroke, patient and caregiver education, regular exercise (e.g. prompting to walk to the toilet, bed exercise for chair-bound patients), abdominal massage, toileting habits, and enteric feeding products containing fiber should be the first-line treatments in non-severe constipation and as adjunctive treatment; even when laxatives are assumed essential for treatment (14, 34-39). Although a better understanding of the etiology and pathophysiology of constipation in each resident is of the physicians’ responsibilities, in daily practice, registered nurses usually provide these treatments rather independently. Thus, nurses are in a key position to develop proactive approaches for preventing and treating the constipation (20). Increased involvement of the nursing staff by physicians and tailoring the treatment for individual residents could probably improve the outcome (12). Despite the limitations of this study such as the relatively small number of participants, the limited geographic area, and the focus only being on one nursing home, our results describe real life in a nursing home and is one of the few studies that has investigated the effectiveness of everyday treatment of constipation in a nursing home. This study was designed to help enhance the comprehension of clinicians and nursing staff regarding the use of laxatives, the factors related to abnormal bowel function, and the effectiveness of laxative treatment among the elderly residents of a nursing home. Treatment of constipation in the nursing home was unsatisfactory. For those who were susceptible to altered bowel function, a coordinated approach is necessary, with involvement of the physicians, the nursing staff, and other professionals including dieticians and pharmacists, to improve the treatment outcome. 

**Table 1.** Characteristics of the Residents and a Comparison Between Residents With Normal and Altered Bowel Function

Characteristics	All Residents, n = 110	Residents With Normal Bowel Function, n = 67	Residents With Altered Bowel Function, n = 43	P Value
**Age, mean (SD), y**	80.7 (7.6)	80.2 (7.1)	81.4 (8.4)	0.39
**Gender, male/female**	55/55	32/35	23/20	0.56
**Body mass index, mean (SD), median [range], kg/m^2^**	21.8 (4.3), 22 [11.7-38.9]	22.7 (4.7), 22.8 [11.7 - 38.9]	20.5 (2.9), 20.5 [16.1 - 29.8]	0.009
**Intake of liquids, mean (SD), median [range], mL/d**	1686.1 (780.4), 1500 [125-3500]	1550.4 (716.3), 1500 [125 - 3500]	1897.7 (836.1), 2000 [500 - 3300]	0.02
**Type of food, regular/soft/ semi-liquid/liquid No. (%)**	40/29/41 (36.4/26.4/37.3)	33/16/18 (49.3/23.9/26.9)	7/13/23 (16.3/30.2/53.5)	0.001
**Length of stay, mean (SD), median [range], mo**	40.7 (25.7), 40.5 [6 - 117]	41.5 (25.7), 40 [7 - 97]	39.5 (25.9), 44 [6 - 117]	0.69
**Barthel Index Score, mean (SD)**	30.8 (32.1)	41.0 (34.5)	13.5 (17.1)	0.000
**MMSE **^**a **^**Score, mean (SD)**	9.1 (10.2)	13.4 (13.3)	3.9 (6.3)	0.000
**Regular physical therapy, No. (%)**	78 (70.9)	48 (71.6)	30 (69.8)	0.83
**Number of diseases, 0/1/2/3, No. (%)**	21/49/30/10 (19.1/44.5/27.3/9.1)	16/31/16/4 (23.9/46.3/13.9/6.0)	5/18/14/6 (11.6/41.9/32.6/14.0)	0.19
**Coronary artery disease, No. (%)**	7 (6.4)	2 (3.0)	5 (11.6)	0.11
**Stroke, No. (%)**	37 (33.6)	17 (25.4)	20 (46.5)	0.03
**Depression Mania, No. (%), cases**	11 (10)	6 (9.0)	5 (11.6)	0.75
**Dementia, No. (%)**	26 (23.6)	14 (20.9)	12 (27.9)	0.49
**Diabetes, No. (%)**	39 (35.5)	26 (38.8)	13 (30.2)	0.36
**Parkinson disease, No. (%)**	11 (10)	6 (9.0)	5 (11.6)	0.75
**ESRD ** ^**a**^ **, No. (%)**	3 (2.7)	2 (3.0)	1 (2.3)	1.00
**SCI ** ^**a**^ **, No. (%)**	5 (4.5)	2 (3.0)	3 (7.0)	0.38
**Number of Medications, less than 5/5 to 8/more than 8 (%)**	42/49/19 (38.2/44.5/17.3)	29/27/11 (43.3/40.3/16.4)	13/22/8 (30.2/51.2/18.6)	0.38
**Antithrombotic agents, No. (%)**	44 (40)	23 (34.3)	21 (48.8)	0.16
**Calcium channel blockers, No. (%)**	47 (42.7)	29 (43.3)	18 (41.9)	1.00
**Diuretics, No. (%)**	33 (30)	18 (26.9)	15 (34.9)	0.40
**Benzodiazepine derivatives, No. (%)**	28 (5.5)	18 (26.9)	10 (23.3)	0.82
**Drugs with anticholinergic effect, No. (%)**	19 (17.2)	9 (13.4)	10 (23.3)	0.20
**Dopaminergic agents, No. (%)**	9 (8.2)	4 (6.0)	5 (11.6)	0.31
**Antidepressants, No. (%)**	10 (9.1)	6 (9.0)	4 (9.3)	1.00
**Analgesics, No. (%)**	7 (6.4)	4 (6.0)	3 (7.0)	1.00

^a^ Abbreviations: ESRD, end stage renal disorder; MMSE, mini mental state examination; SCI, spinal cord injury.

**Table 2.** Dosage Schedules of the Laxatives

Substance	ATC ^a^-Level 5	Dosage Schedules, No. (%)		
		On demand	Regular Use of Standard Dose	Regular Use of High Dose
**Bisacodyl**	A06AB02	0	24 (21.8)	1 (0.9)
**Senna glycoside**	A06AB06	0	65 (59.1)	1 (0.9)
**Sterculia**	A06AC03	0	3 (2.7)	1 (0.9)
**Lactulose**	A06AD11	2 (1.8)	2 (1.8)	1 (0.9)
**Magnesium oxide**	A06AD02	0	31 (28.2)	20 (18.2)
**Bisacodyl**	A06AG02	1 (0.9)	3 (2.7)	0
**Glycerol**	A06AG04	47 (42.7)	31 (28.2)	0

^a^ Abbreviation: ATC, anatomical therapeutic chemical classification system.

**Table 3.** Bowel Function Related to Efficacy of the Laxative Regimen ^a^

Laxatives	Normal Bowel Function, n = 67	Altered Bowel Function, n = 43
**No record of use or only enema on demand, No. (%)**	13 (72.2%)	5 (27.8%)
**One regular laxative, No. (%)**	26 (74.3%)	9 (25.7%)
**Two regular laxatives, No. (%)**	21 (58.3%)	15 (41.7%)
**More than two regular laxatives, No. (%)**	7 (33.3%)	14 (66.7%)

^a^ There were significant differences between the groups (exact chi-square P = 0.015; and linear-by-linear P = 0.004).
